# The What and Why of Research on Reinforcement

**DOI:** 10.1371/journal.pbio.0020420

**Published:** 2004-12-14

**Authors:** Maria R Servedio

## Abstract

Reinforcement - a process that helps prevent interbreeding between hybridising populations - is an important and little understood mechanism driving the completion of speciation

Reinforcement, like sympatric speciation (see Box 1), has charisma. Evolutionary biologists are still deeply uncertain about how often these processes take place, and hence how important they are in explaining the biological diversity we see today. Empirical and theoretical support for both ideas has waxed and waned over recent decades. Yet both ideas have consistently garnered an unusual amount of attention.

**Box 1. Glossary**

**Sympatry**—Area(s) of overlap in the ranges of populations, enabling potential interbreeding.

**Allopatry**—Area(s) of population ranges that do not overlap with one another, preventing interbreeding.

**Sympatric speciation**—Speciation that occurs within a range of sympatry.

**Reinforcement**—The evolution of mechanisms that prevent interbreeding between newly interacting incipient species, as a result of selection against hybrids (narrow definition) or interspecific matings (broad definition) (See Figure 1).

**Conspecific sperm precedence**—Disproportional fertilization of a female by sperm of a conspecific male, when that female has mated with both conspecific and heterospecific males.

Much of the appeal of both reinforcement and sympatric speciation lies in the way they unite micro- and macroevolution. Reinforcement, a concept popularized by Dobzhansky (1937), is a process by which speciation, a macroevolutionary process, can be driven directly by natural selection, one of the primary microevolutionary forces. Sympatric speciation can make the same claim. Because of this close linkage between the concepts, the study of one can tell us a great deal about the other (see Kirkpatrick and Ravigné 2002). Such studies can also reveal a lot about the general role of microevolution in species divergence.

Reinforcement provides a pathway toward the completion of the speciation process. Imagine that two divergent populations (potentially even classified as separate species) come into contact after a period of allopatry (Figure 1). If the populations have been apart for a long time, evolved differences between them will cause a certain degree of incompatibility when the populations come together. Often, this incompatibility comes in the form of low hybrid fitness (postzygotic isolation) or mismatched mating characteristics (premating isolation). The degree of the development of these isolating mechanisms is roughly proportional to the genetic distance between the populations, reflecting the fact that incompatibilities accumulate over time (Coyne and Orr 1989).

**Figure 1:**
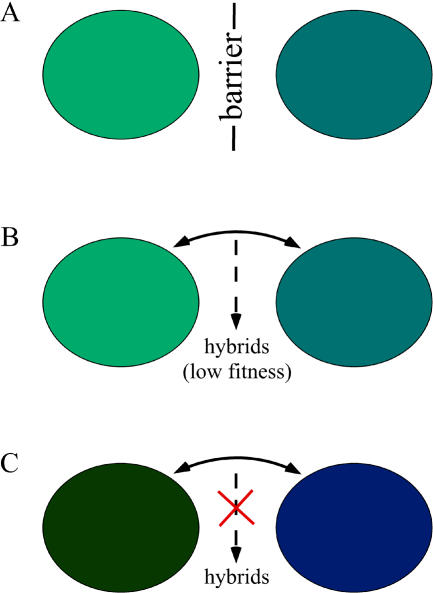
Schematic Diagram of Reinforcement (A) Populations diverge by evolving separately for a period of time in allopatry (separated by a geographic barrier). Populations may still be completely compatible in their mating characteristics, or these may also have diverged slightly (represented in the figure by the differences in color). (B) Secondary contact commences. This is shown through the removal of a geographic barrier that allows individuals to migrate between populations (migration is represented by a bidirectional arrow). Secondary contact can also occur by range expansion to produce an area of sympatry, or through other similar mechanisms. Due to the prior divergence between populations, hybrids have low fitness. (C) Selection to avoid producing low fitness hybrids causes the evolution of further divergence in the mating traits (represented by color) of the two populations, reducing interbreeding.

If the isolating mechanisms between these populations are only partially complete, extensive hybridization may occur. This can result in fusion back into a single population, or in the swamping of one population's gene pool by the genes of the other (extinction). But there is another possibility, one that can cause the speciation between the two populations to proceed. Remember that if the populations have been separated for long enough, it is likely that hybrids between them will have relatively low fitness. Individuals who mate with members of the opposing population will therefore produce offspring of poor quality, and hence have lower fitness than individuals that mate within their own population. This favors the evolution (or further divergence) of characteristics that cause mating within, rather than between, populations (Figure 1C). Speciation between the populations is driven further towards completion through this increase in premating isolation.

This process, the evolution of premating isolation after secondary contact due to selection against hybrids, is reinforcement sensu Dobzhansky (1937). Recent authors have broadened the definition of reinforcement to include as a driving force any form of selection against mating between populations (e.g., Servedio and Noor 2003). This could include, for example, lower fertility, or higher mortality of females that mate with members of other populations. In all definitions, however, the microevolutionary process of selection is essential for reinforcement. In fact, in reinforcement, speciation itself can be thought of as an adaptive response to selection. It is little wonder that this causal linking of micro- and macroevolution has appeal for many evolutionary biologists.

## Reinforcement in the 21st Century

Despite the substantial progress in our understanding of reinforcement that has been achieved over the last few decades, many questions remain about the process. These questions lend themselves to exploration by a broad variety of disciplines (evolution, ecology, behavior, phylogenetics, phylogeography, genetics), approaches (experimental, observational, comparative, theoretical) and taxonomic systems.

**Figure 2:**
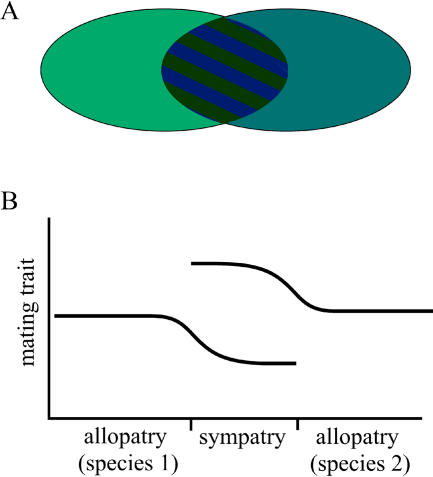
The Pattern of Reproductive Character Displacement (A) Reproductive character displacement due to the presence of an area of overlap between two populations (differences in mating characteristics are represented by color changes, with the hatched area showing divergence in sympatry). Reproductive character displacement can also occur when the sympatric and allopatric areas are not contiguous. (B) Reproductive character displacement can appear as a cline in mating cues or mating preferences (y-axis), with divergence originating in the area of sympatry and spreading into areas of allopatry.

Doubtless, the most important unanswered question about reinforcement is how often it occurs. It is very difficult to prove that reinforcement is occurring, or has occurred, between two species. Reinforcement occasionally leaves a signature, called reproductive character displacement, in which mating characteristics have diverged between populations in areas of sympatry but not areas of allopatry (Figure 2) (the relationship between reinforcement and reproductive character displacement, and controversy over the definition of the latter, is reviewed in Howard 1993). In sympatric areas, populations are capable of producing hybrids, which drives reinforcement, while in allopatry hybrid production, and hence the selection for reinforcement, is absent. Reproductive character displacement has been found to be common, suggesting to some that reinforcement may be common as well (Howard 1993). It is universally acknowledged, however, both that reproductive character displacement can be caused by processes other than reinforcement, and that reinforcement can occur without leaving this signature (e.g., when population ranges are completely sympatric). Proving that reinforcement has occurred requires the ruling out of several alternative hypotheses, which are themselves difficult to assess (Noor 1999; Coyne and Orr 2004).

Several isolated examples of reinforcement between specific pairs of species have been demonstrated, fairly conclusively, in a variety of taxa including Drosophila pseudoobscura and D. persimilis (Noor 1995), flycatchers (Sætre et al. 1997), sticklebacks (e.g., Rundle and Schluter 1998), spadefoot toads (Pfennig 2003), and walking-stick insects (Nosil et al. 2003) (see also reviews of Noor 1999; Coyne and Orr 2004). These studies involve a variety of behavioral tests of mate choice, analyses of hybrid fitness and the production of hybrids in the wild, and controls for alternative explanations.

While examples such as these provide essential information about reinforcement, their slow rate of compilation and biased reporting do not provide efficient ways to assess how often reinforcement occurs in general. Comparative approaches, which examine patterns across a broader taxonomic group, can also provide support for reinforcement without these detailed mechanistic analyses (review in Coyne and Orr 2004). The revival of reinforcement in the late 1980s began with one such study in the genus Drosophila (Coyne and Orr 1989). By comparing patterns across a wide number of species, such studies can give a better assessment of the potential frequency with which reinforcement occurs—without, however, providing conclusive evidence for reinforcement between specific species pairs.

Another area where further research is essential is the determination of which biological factors promote reinforcement, as opposed to population fusion. Theoretical studies, using mathematical models and computer simulations, are proving useful in pinpointing the effects of many factors such as migration rates and patterns, the type of selection against interspecific mating, and the genetic basis of premating isolation (reviews in Turelli et al. 2001; Servedio and Noor 2003). Fortunately, some of the cases of reinforcement in specific species pairs are now being developed to the point where they can address similar questions (e.g., sex linkage of mating genes; Sætre et al. 2003). Both theoretical studies and these well developed empirical systems are also starting to address a third important area of research: how reinforcement interacts with other forces, such as ecological selection pressures, that promote speciation (e.g., Servedio 2004; Nosil et al. 2003). These integrated studies are essential to the correct placement of reinforcement within the bigger context of speciation processes.

In recent years, exciting developments have started to take place in the analysis of the genetics of reinforcement (reviewed in Servedio and Noor 2003). These developments both parallel and overlap with progress made on the genetics of speciation and species differences in general. For example, significant progress has recently been made in identifying the genetic control of hybrid incompatibilities (e.g., Presgraves et al. 2003; Barbash et al. 2003). This progress has been accompanied by a new understanding of how chromosomal rearrangements may allow these incompatibilities to be maintained despite hybridization in sympatry (Rieseberg 2001; Navarro and Barton 2003; Brown et al. 2004). Sympatric maintenance of incompatibilities, of course, has profound implications for reinforcement, which requires these incompatibilities as the force driving divergence (Noor et al. 2001).

Genetic analysis is also allowing a new understanding of the mechanisms by which reinforcement might be taking place in specific cases. Work by Ortiz-Barrientos et al. (2004) in this issue of *PLoS Biology* illustrates the extent of the insights that can be made with this approach. Using high-resolution genetic mapping the authors have identified the locations of genes that cause increased discrimination against Drosophila persimilis males by D. pseudoobscura females, due to reinforcement in sympatry. Surprisingly, these genes map to very different areas of the chromosomes than do genes that cause a basal level of mating discrimination between the species in allopatry. Among other insights, the position of these genes suggests that the reinforced discrimination is based on odor, not on the mechanism used in allopatry, male song. This leads to the novel conclusion that reinforcement is not just increasing the strength of an already existing mechanism of species discrimination, but is occurring through the development of a new discrimination system. These kinds of developments can also motivate more realistic theoretical models of the reinforcement process.

## Implications and Extensions of Reinforcement

What if, when our assessment of the frequency of reinforcement is improved, it turns out to have been a rare occurrence in the generation of current biological diversity? The study of reinforcement is broad and varied enough that many of our findings about the process would still have wide-reaching implications.

First, recall the claim, at the start of this article, that studying reinforcement reveals much about the role of microevolution in the macroevolutionary process of speciation. Knowledge gained about this relationship is not only directly applicable to the very similar process of sympatric speciation, but can also tell us a great deal about speciation caused by ecological adaptation and sexual selection, which are critical components of reinforcement in many systems (e.g., Nosil et al. 2003; Haavie et al. 2004). Studies looking for reinforcement have also led to insights into the formation and maintenance of hybrid zones (e.g., Butlin 1998; Britch et al. 2001). Situations where reinforcement fails to occur likewise teach a lesson, elucidating possible mechanisms of extinction when secondary contact occurs between species.

Second, analysis of reinforcement clarifies the interactions between levels of reproductive isolation that occur at different stages in the life cycle. Reinforcement, broadly defined, can be driven by isolation at the postzygotic level or by incompatibilities that occur between mating and zygote production (postmating-prezygotic incompatibilities; Servedio 2001). Postzygotic isolation can likewise cause divergence at the premating stage (reinforcement) or potentially at the postmating-prezygotic stage, through the evolution of conspecific sperm precedence (Marshall et al. 2002). These various stages of isolation have different degrees of importance among plants, free-spawning marine invertebrates, and other internally and externally fertilizing animals (Bernasconi et al. 2004). Analysis of these stages of isolation, their interactions, and the evolutionary pressures they are under therefore has broad implications for comparative reproductive biology across these varied groups.

Finally, regardless of whether reinforcement has been a common pathway in speciation, its relevance may be increasing. Reinforcement is a possible outcome anytime species that are capable of hybridization come into contact. Human activity is increasing the incidence of secondary contact by altering habitat and introducing invasive species. This contact often results in hybridization (reviews in Rhymer and Simberloff 1996; Mooney and Cleland 2001). It is important to identify and understand the properties of species pairs that make extensive introgression, extinction, stable hybrid zones, or reinforcement likely outcomes of such contact. If reinforcement has played a small role in the generation of current diversity, it may be because secondary contact itself has historically been a rare occurrence. It is the frequency of reinforcement among incidences of secondary contact that will determine its importance in the near future.
